# Adult height in pubertal boys with short stature treated with GH/letrozole: a hospital record-based retrospective study

**DOI:** 10.1186/s12887-022-03438-4

**Published:** 2022-06-28

**Authors:** Yaping Ma, Ruofan Jia, Bingyang Xia, Bin Tang, Zhuangjian Xu

**Affiliations:** Department of Pediatrics, Affiliated Hospital of Jiangnan University, Wuxi, 214122 China

**Keywords:** Pubertal boys, Short stature, Letrozole, Growth hormone, Adult height

## Abstract

**Background:**

The growth potential in pubertal boys with short stature is limited by the effect of estrogen on epiphyseal fusion. This study aims to identify the efficacy and safety of the combination of growth hormone (GH) and letrozole on adult height (AH) in pubertal boys with short stature.

**Methods:**

This is a retrospective record based study. Pubertal boys with short stature who were treated with GH and letrozole were followed up at outpatient clinics in our hospital. Twenty subjects who reached AH are reported here.

**Results:**

Baseline chronological age was 12.12 ± 1.14 yr and bone age was 13.00 ± 0.93 yr. The period of GH/letrozole treatment was 1.94 ± 0.67 yr. Height standard deviation score for bone age was increased from -1.46 ± 0.51 before treatment to -0.12 ± 0.57 after treatment (*P* < 0.001). The predicted AH before treatment, predicted AH after treatment, AH, and genetic target height were 161.02 ± 4.12 cm, 172.11 ± 4.20 cm, 172.67 ± 2.72 cm, and 167.67 ± 3.56 cm, respectively. There was a significant predicted AH difference before and after treatment (*P* < 0.001). There was a significant difference between predicted AH before treatment and genetic target height (*P* < 0.001). Predicted AH after therapy was higher than that of gene target height (*P* < 0.001), as well as AH and genetic target height (*P* < 0.001). There was no significant side effect.

**Conclusions:**

GH and letrozole combination can enhance AH in pubertal boys with short stature.

## Introduction

In recent years, how to improve the adult height (AH) of pubertal boys with short stature has drawn more attention in pediatric endocrinology and related fields. As it is known about the close relationship between estrogen and bone age, high estrogen levels in puberty may lead to premature epiphyseal closure, which causes AH unsatisfied. Gonadotropin-releasing hormone analog (GnRHa), an effective drug for decreasing estrogen levels, is approved to inhibit the hypothalamic-pituitary–gonadal axis (HPGA) in children. GnRHa is used for boys whose bone ages are younger than 12.5 years old, and it should be cautious for boys older than 12.5 years old of bone age [1]. When bone age is above 13.5 years old, the effect of GnRHa treatment alone on improving AH is not significant. So pubertal boys with short stature and accelerated bone age are not suitable to use GnRHa treatment only.

Letrozole is one of the third-generation aromatase inhibitors, which can inhibit estrogen generation more than 99.1% [2]. Its primary clinical indication is cancer, especially hormone receptor-positive early breast cancer and late breast cancer in postmenopausal women [3]. Currently, the words “can be used for children” have not been found in the instructions of letrozole. However, recently more studies on letrozole have been carried out in pediatrics, especially in pediatric endocrine clinical application. Specifically, it has been applied in some endocrine diseases of children, such as gynecomastia, growth hormone (GH) deficiency, idiopathic short stature, and congenital adrenal hyperplasia [4–7]. Compared to GnRHa, letrozole only inhibits estrogen generation, but does not affect the child's normal development. It means that letrozole can inhibit the increase of bone age and at the same time allow the children to have normal pubertal psychology. Recently, the clinical application of GH/letrozole combination in treating pubertal boys with short stature has gradually increased. However, few reports were focused on the cases that reached AH after GH/letrozole combination treatment. Twenty subjects who received the combination treatment and reached AH are reported here.

## Methods

### Subjects

This study was approved by the Ethics Committee of Affiliated Hospital of Jiangnan University. Informed consent was obtained from patients’ parents before treatment. We set up the following groups of treatments: anastrozole alone, letrozole alone, GH/anastrozole combination, GH/letrozole combination, and parents can voluntarily choose one of the treatments. From August 2009 to December 2020, we followed up 80 subjects who received GH/letrozole combination treatment at outpatient clinics, among which twenty subjects reached AH. Given that the case number in the other three groups was minimal and no cases reached AH during the follow-up period, we do not report them here.

The referenced diagnostic criteria are as follows. Normal puberty is defined as gonadal development that begins after nine years, with testis volume > 4 mL. There is evidence for activated HPGA from gonadotropin-releasing hormone (GnRH) stimulation test or single serum gonadotropin level [1]. Short stature and short stature of predicted AH (PAH) are defined as height or PAH below the third percentile of the growth curve of healthy children of the same age and gender [8]. AH is defined as epiphyseal closure on hand-wrist bone age X-ray and no changes (< 1 cm) in height for 12 months [9]. The inclusion criteria: boys, normal puberty with activated HPGA and short stature (or short stature of PAH) and bone age < 16 yr. The exclusion criteria: prepuberty, asymmetric short stature (such as achondroplasia), severe acute and chronic liver or kidney diseases, and tumors. The criteria for stopping the GH/letrozole treatment are: PAH is satisfied, and both genetic target height (THt) and growth velocity per year (< 2 cm/year) need to be considered simultaneously.

### Examination, treatment, and monitoring

Before treatment, HPGA was evaluated and shown to be activated. Physical measurements were taken, such as weight, height, and testicular development. Blood biochemical indexes related to liver function, kidney function and electrolyte, glycometabolism indexes (such as glycosylated hemoglobin, oral glucose tolerance test, insulin release test), IGF-1, IGF-BP3, tumor indexes (such as alpha-fetoprotein and chorionic gonadotropin), pituitary gland magnetic resonance imaging, B ultrasound of the upper abdomen, thyroids and adrenal gland, and bone age were evaluated. After evaluation, cases were treated with GH (0.15–0.20 IU/kg·d, subcutaneous injection) and letrozole tablets (0.8–2.5 mg/day, oral). We adjusted GH dosage according to IGF-1 levels. The dosage of letrozole was adjusted according to testosterone and estradiol levels. During the treatment period, the indicators of physical measurement, blood biochemistry, glucose metabolism, IGF-1, IGF-BP3, and bone age were monitored regularly, and subjects were routinely supplemented with vitamin D and calcium tablets.

THt is calculated as: (sum of parents’ height + 13)/2 ± 5 cm.

### Lab test

An automatic biochemical analyzer assayed biochemical blood indexes and glycometabolism indexes (HITACHI7170A, Japan). The IGF-1, IGF-BP3, tumor indexes and sex hormones were measured by ACCESS immunochemiluminescence analyzer (DXI800, USA).

### Statistical analysis

SPSS19.0 was used to analyze the data. Paired *t-*test and Wilcoxon Signed-Rank test were used to compare the data before and after treatment. A two-tailed *P* value < 0.05 was considered to be statistically significant.

## Results

### Baseline characteristics

At the beginning of the study, chronological and bone ages were 12.12 ± 1.14 yr and 13.00 ± 0.93 yr, respectively. Height and body mass indexes were 148.20 ± 6.87 cm and 19.78 ± 3.05 kg/m^2^, respectively. Nine boys were at Tanner stage 2, three at Tanner stage 3, six at Tanner stage 4, and two at Tanner stage 5 (Table 1). Subjects 1 and 2 were twins, both of whom were obese and had insulin resistance but with normal blood glucose. They took metformin about two months before GH/letrozole treatment. Subjects 1 and 2 took metformin during the treatment until the end of treatment. At the end of treatment, their body mass index returned to normal.

**Table 1:** Clinical and laboratory data of 20 pubertal boys with short stature before GH/Letrozole treatment

Subject No.	Age-ranges (y)	BMI (kg/m2)	Height SDS for CA	Bone age(y)	Height SDS for bone age	Testis volume(ml)	Tanner stage	Testosterone(ng/ml)	Estradiol (pg/ml)	Target height (cm)
1	11–14	25.8	-1.33	13.9	-2.37	25.0	5	2.08	21.00	166.5
2	11–14	26.2	0.33	14.4	-1.42	25.0	5	3.22	25.00	166.5
3	9–12	19.5	0.32	13.4	-1.83	10.0	3	2.16	26.00	165.8
4	11–14	15.7	-2.64	12.9	-2.42	6.0	2	0.77	39.00	168.5
5	9–12	17.7	-0.02	12.3	-1.28	16.0	4	4.96	54.00	165.0
6	10–13	20.4	-0.97	12.6	-1.66	8.0	2	0.58	6.00	167.5
7	10–13	18.9	-0.95	12.4	-1.49	6.5	2	0.93	1.00	173.0
8	10–13	19.1	-0.92	12.7	-1.36	8.0	2	3.51	69.00	169.0
9	10–13	23.6	0.54	12.8	-0.55	15.0	4	1.59	7.00	167.5
10	9–12	19.5	0.91	13.1	-0.87	18.0	4	0.48	17.28	168.0
11	9–12	21.8	-0.48	12.4	-1.47	4.5	2	1.61	30.00	165.0
12	12–15	17.4	0.26	14.1	-1.01	20.0	4	5.33	25.00	166.0
13	11–14	18.1	-0.79	13.2	-1.58	21.0	4	5.20	9.00	161.2
14	9–12	17.6	0.27	13.0	-1.62	10.5	3	1.91	13.00	176.4
15	12–15	16.8	0.14	15.1	-1.83	10.0	3	4.29	18.30	171.3
16	10–13	20.0	-1.31	12.1	-1.51	8.0	2	0.62	3.00	169.7
17	11–14	23.3	-0.08	13.4	-1.13	8.0	2	2.04	19.00	165.5
18	9–12	15.1	-1.58	10.8	-1.45	4.0	2	0.19	9.00	167.5
19	9–12	20.4	-0.41	12.3	-1.88	6.0	2	0.22	26.00	162.0
20	11–14	18.6	0.09	13.0	-0.39	18.0	4	1.64	17.00	171.5

*GH* Growth hormone, *No.* Number, *SDS* Standard deviation score, *BMI* body mass index, *CA* Chronological age

The treatment duration was 1.94 ± 0.67 yr (0.9 ~ 3.2 yr). At the end of the treatment, the chronological and bone ages were 14.06 ± 1.28 yr (11.9 ~ 16.0 yr) and 14.41 ± 1.31 yr respectively. Height and body mass indexes were 165.53 ± 4.10 cm and 21.80 ± 2.73 kg/m^2^ respectively. Growth velocity was 9.34 ± 2.28 cm per year. Subjects 9 and 19, who had normal body mass index before treatment, became obese at the end of treatment. At the end of treatment, all boys were at Tanner stage 4 or 5 (Table 2).

**Table 2:** Clinical and laboratory data of 20 pubertal boys with short stature after GH/Letrozole treatment

Subject No.	Age-ranges (y)	BMI (kg/m2)	Height SDS for CA	Bone age(y)	Height SDS for bone age	Testis volume(ml)	Tanner stage	Testosterone (ng/ml)	Estradiol (pg/ml)	Growth velocity (cm/y)	Final height (cm)
1	13–16	26.5	0.75	14.2	-0.59	25.0	5	6.74	1.00	6.9	170.0
2	13–16	24.1	2.38	15.7	0.06	25.0	5	3.54	0.00	6.4	174.0
3	10–13	21.1	1.10	14.5	-1.30	20.0	4	7.14	16.00	11.9	172.5
4	14–17	18.8	0.83	13.4	0.08	25.0	5	7.25	23.00	10.3	175.0
5	11–14	20.7	1.12	14.6	-1.01	15.0	4	6.87	10.00	9.3	172.0
6	13–16	21.7	1.67	16.2	-0.69	21.0	5	11.28	0.00	7.5	170.0
7	12–15	17.9	2.07	14.7	0.01	20.0	4	2.13	5.00	11.4	172.5
8	12–15	23.3	1.80	14.1	0.17	15.0	4	7.16	17.00	10.6	174.5
9	11–14	25.6	1.76	13.5	0.46	21.0	4	7.10	0.00	11.4	175.0
10	10–13	20.3	1.97	13.9	-0.10	18.0	4	一 ^a^	一 ^a^	12.6	172.0
11	11–14	23.0	1.57	12.9	0.84	25.0	5	10.19	14.00	10.8	170.6
12	14–17	17.9	1.55	15.4	-0.60	20.0	4	4.46	12.00	6.2	173.0
13	13–16	21.4	1.38	14.3	-0.20	25.0	5	3.22	12.00	6.3	167.5
14	10–13	19.1	1.88	14.0	-0.10	25.0	5	7.14	0.00	12.1	173.2
15	14–17	19.9	1.80	18.3	-0.69	15.0	4	10.39	36.00	5.5	169.0
16	13–16	25.1	1.63	14.0	0.14	25.0	5	7.60	8.00	9.8	173.5
17	12–15	23.9	1.97	14.9	-0.08	20.0	4	7.79	5.00	10.9	178.0
18	12–15	19.2	-0.49	12.1	0.15	15.0	4	5.81	14.00	7.4	170.0
19	12–15	26.0	1.55	13.9	0.06	25.0	5	6.57	39.00	8.6	173.0
20	13–16	20.4	2.29	13.5	0.99	25.0	5	6.96	35.00	10.8	178.0

*GH* Growth hormone, *No.* number, *SDS* Standard deviation score, *BMI* body mass index, *CA* Chronological age

^a^Data missing

### Comparison of indexes

Serum estradiol was decreased from 21.729 ± 16.870 pg/ml before treatment to 13.000 ± 12.472 pg/ml after treatment (*Z* = -2.054, *P* = 0.040). Testosterone was increased from 2.167 ± 1.692 ng/ml before treatment to 6.807 ± 2.339 ng/ml after treatment (*Z* = -2.213, *P* = 0.027). After treatment, the range of testosterone was 2.13–11.28 ng/ml, and no hypertestosteronemia was observed [10] (Tables 1 and 2).

After the treatment, height increased from 148.20 ± 6.87 cm to 165.53 ± 4.10 cm (*t* = -14.012, *P* < 0.001). Height standard deviation score (SDS) for bone age increased from -1.46 ± 0.51 to -0.12 ± 0.57 (*Z* = -3.921, *P* < 0.001). Height SDS for chronological age increased from -0.43 ± 0.87 to 1.53 ± 0.64 (*t* = -12.007, *P* < 0.001). The PAH before treatment (PAH1), PAH after treatment (PAH2), AH, and THt were 161.02 ± 4.12 cm, 172.11 ± 4.20 cm, 172.67 ± 2.72 cm, and 167.67 ± 3.56 cm, respectively. There was a significant difference between PAH1 and PAH2 (*t* = -10.831, *P* < 0.001), as well as PAH1 and THt (*t* = -5.814, *P* < 0.001). PAH2 was significantly higher than THt (*t* = -3.940, *P* < 0.001). In addition, AH is significantly higher than THt (*t* = 5.714, *P* < 0.001). The PAH1 minus THt (PAH1-THt), PAH2 minus THt (PAH2-THt), and AH minus THt (AH-THt) were -6.65 ± 5.11 cm, 4.44 ± 5.03 cm, and 5.00 ± 3.91 cm, respectively. There were significant differences between PAH1-THt and PAH2-THt (*t* = -10.831, *P* < 0.001), as well as PAH1-THt and AH-THt (*t* = -13.372, *P* < 0.001). No difference between PAH2-THt and AH-THt (*t* = -0.625, *P* = 0.540) was seen (Tables 1 and 2).

### Safety and tolerability

One subject had a single ankle and metatarsal fracture during the treatment. Later, it was found out that the boy refused to take vitamin D and calcium tablets. One subject had scoliosis at the end of treatment (21 degrees, no anterior and lateral spine radiographs were taken before treatment). Two subjects developed hypothyroxinemia but recovered after treatment. Two subjects had insulin resistance before treatment, but did not deteriorate during the treatment, and recovered after the treatment. Another 5 subjects had insulin resistance during the course of treatment, but all recovered after the treatment.

## Discussion

One of the big issues faced by pubertal boys with short stature is the limited growth potential. Our treatment goal is to explore their residual growth ability and to help them reach satisfied height (close to THt). At present, there is still little literature on the treatment of adolescent short boys with GH combined with letrozole who were followed up until they reached AH. Mauras and colleagues treated severe pubertal idiopathic short stature with GH (0.13U/kg·d), or aromatase inhibitor (anastrozole or letrozole)/GH combination for the next 24 months [6]. There were 12–13 cases treated with GH/letrozole combination in that study, but it was not clear whether the subjects reached AH after the treatment. In our study, the treatment duration was 1.94 ± 0.67 yr. We saw 20 cases out of 80 GH/letrozole combination cases reached AH after similar treatment. Our GH dosage (0.15–0.2U/kg·d) was slightly higher than that used in Mauras’ study, but the dose was still within the recommended guidelines on GH usage [11]. Mauras's study's mean height gain at 24 months was 18.9 ± 0.8 cm, and the absolute change in height was 22.5 ± 1.4 cm [6]. Our mean height gain was 17.34 ± 5.53 cm. If calculated by 24 months, the net height gain would be 17.9 cm, which would be close to the 24 months height gain (18.9 cm) in Mauras’s study. Our net height gain was 24.47 ± 6.70 cm, which was close to Mauras’ study (22.5 ± 1.4 cm). What’s more, the endpoint of our study was AH. In our study, the effect of GH combined with letrozole on AH was observed to avoid the interference of mixed factors of letrozole and anastrozole. Because they have different inhibitory rates on estrogen (letrozole > 99.1%, anastrozole 97.3%) [2].

Recently, Pedrosa et al. reported that 96 adolescent boys were treated with anastrozole, GH combined with anastrozole, letrozole, or GH combined with letrozole. They concluded that GH combined with letrozole could maximize near AH [12]. There were 22 near AH cases but no AH cases in that study [12]. We compared the data of 9 near AH cases treated with GH/letrozole combination in Pedrosa’s study with our data. There is no significant difference in pre-treatment bone age (13.28 ± 1.09 yr v.s.13.00 ± 0.93 yr, *Z* = -1.182, *P* = 0.237) and height (152.17 ± 6.99 cm v.s. 148.20 ± 6.87 cm, *t* = 1.432, *P* = 0.163)] between our study and Pedrosa’s study. Although our study had lower before treatment PAH1 (161.02 ± 4.12 cm v.s. 170.27 ± 2.35 cm, *t* = 6.251, *P* < 0.001) and THt (167.67 ± 3.56 cm v.s. 172.89 ± 4.53 cm, *t* = 3.357, *P* = 0.002) compared to those in Pedrosa’s study, there was no significant difference between near AH in the Pedrosa’s study and AH in our study (174.58 ± 7.01 cm v.s. 172.67 ± 2.72 cm, *t* = 0.793, *P* = 0.448). Thus, it strongly suggests similar GH/letrozole treatment efficacy on pubertal boys with short stature.

It is known that letrozole can significantly inhibit bone age progress and improve AH in adolescent short boys. In a prospective, double-blind, randomized, placebo-controlled clinical study, thirty-one idiopathic short boys aged 9.0–14.5 yr were treated with the letrozole (2.5 mg/d) for 2 yr. The serum testosterone level in the letrozole group was significantly higher than that in the control group. After two years of treatment, the range of serum testosterone entering puberty was 17.3–1385 ng/dl, while serum estradiol level was similar to that before treatment [13]. In our study, testosterone (6.807 ± 2.339 ng/ml, 2.13 to 11.28 ng/ml) after treatment was higher than that of before treatment (2.167  ±1.692 ng/ml, 0.19 to 5.33 ng/ml). Serum estradiol level (13.000 ± 12.472 pg/ml) was significantly decreased compared to that before treatment (21.729 ± 16.870 pg/ml). The bone age minus chronological age decreased from 0.88 ± 0.83 to 0.35 ± 1.34 (*t* = 2.785, *P* = 0.012). It strongly suggests that letrozole prevented the conversion of testosterone to estradiol, promoted the slow growth of bone age, and gradually shortened the bone age minus chronological age. It provided the opportunity for pubertal boys with short stature to reach AH with serum testosterone in the normal range.

It has been found that GH combined with GnRHa did not improve AH in adolescent short boys. Thirty-two short adolescents with Tanner stage 2–3, age and bone age less than 12 yr for girls or less than 13 yr for boys, were randomly allocated to receive GH combined GnRHa (n = 17) or no treatment (n = 15) for 3 yr. AH was assessed at 18 yr or older in girls or 19 yr or older in boys. There was no AH difference between the treatment and control groups. Mean lumbar spine bone mineral density and bone mineral apparent density tended to be lower in treated boys. GH combined GnRHa can not be considered a routine treatment for boys with short stature in adolescent [14].

Among the common side effects of GH, scoliosis progression during GH treatment appears due to rapid growth rather than as a direct side effect of GH. Scoliosis is observed in ∼0.2% of children with idiopathic short stature or idiopathic GH deficiency treated with GH [15, 16]. In our study, there was a case of scoliosis with a growth rate of 10.9 cm/year. Unfortunately, no anteroposterior and lateral spine radiographs were taken from the child before treatment. We think it is likely that the growth rate of scoliosis is related to rapid growth.

In addition to the common side effects of GH, many scholars have paid attention to the side effects of letrozole in short-stature boys, but there is some controversy. In a survey in 2015, a significant proportion (45%) of prepubertal boys with idiopathic short stature treated with letrozole developed mild morphological abnormalities of their vertebrae, compared with none in the placebo group [17]. The incidence of vertebral deformation in children with idiopathic short stature increased after letrozole was used. Still, there was a similar incidence between the letrozole and the control groups, so there may be other reasons for the vertebral deformation [18]. Vertebral deformities do not occur in boys with delayed puberty treated with letrozole for one year [19]. No cases with abnormal vertebral morphology were found in our study. Therefore, the effects of letrozole on vertebral deformation need further study.

Letrozole does not adversely affect insulin sensitivity in peripubertal boys [20]. In our study, subjects 1 and 2 had insulin resistance. To ensure blood insulin homogeneity before enrollment and not affect the use of GH, they took metformin two months before and through the treatment of GH/letrozole. Insulin resistance did not deteriorate during the treatment and recovered after the treatment. Another 5 subjects had insulin resistance during the treatment, but all recovered after the treatment.

## Conclusions

GH combined with letrozole can improve the final residual growth ability of pubertal boys with short stature and help them reach AH. The effects of letrozole on vertebral deformation need further study.

## Data Availability

The datasets generated and/or analysed during the current study are not publicly available due to that the data owner is the Affiliated Hospital of Jiangnan University but are available from the corresponding author on reasonable request.

## Abbreviations

GH: Growth hormone
AH: Adult height
GnRHa: Gonadotropin-releasing hormone analog
HPGA: Hypothalamic-pituitary–gonadal axis
GnRH: Gonadotropin-releasing hormone
PAH: Predicted adult height
THt: Target height
SDS: Standard deviation score
PAH1: PAH before treatment
PAH2: PAH after treatment
PAH1-THt: PAH1 minus THt
PAH2-THt: PAH2 minus THt
AH-THt: AH minus THt
